# Impact of temperature on the affinity of SARS-CoV-2 Spike glycoprotein for host ACE2

**DOI:** 10.1016/j.jbc.2021.101151

**Published:** 2021-08-31

**Authors:** Jérémie Prévost, Jonathan Richard, Romain Gasser, Shilei Ding, Clément Fage, Sai Priya Anand, Damien Adam, Natasha Gupta Vergara, Alexandra Tauzin, Mehdi Benlarbi, Shang Yu Gong, Guillaume Goyette, Anik Privé, Sandrine Moreira, Hugues Charest, Michel Roger, Walther Mothes, Marzena Pazgier, Emmanuelle Brochiero, Guy Boivin, Cameron F. Abrams, Arne Schön, Andrés Finzi

**Affiliations:** 1 Centre de Recherche du CHUM, axe Immunopathologie, Montreal, Quebec, Canada; 2 Département de Microbiologie, Infectiologie et Immunologie, Université de Montréal, Montreal, Quebec, Canada; 3 Centre de Recherche du CHU de Québec, Université Laval, Quebec City, Quebec, Canada; 4 Department of Microbiology and Immunology, McGill University, Montreal, Quebec, Canada; 5 Département de Médicine, Université de Montréal, Montréal, Quebec, Canada; 6 Department of Biochemistry and Molecular Biology, Drexel University College of Medicine, Philadelphia, Pennsylvania, USA; 7 Laboratoire de Santé Publique du Québec, Institut Nationale de Santé Publique du Québec, Sainte-Anne-de-Bellevue, Quebec, Canada; 8 Department of Microbial Pathogenesis, Yale University School of Medicine, New Haven, Connecticut, USA; 9 Infectious Disease Division, Department of Medicine, Uniformed Services University of the Health Sciences, Bethesda, Maryland, USA; 10 Department of Biology, The Johns Hopkins University, Baltimore, Maryland, USA

**Keywords:** coronavirus, COVID-19, SARS-CoV-2, Spike glycoproteins, RBD, ACE2, temperature, N501Y, neutralization, variants of concern, ACE2, angiotensin-converting enzyme 2, AEC, airway epithelial cell, BLI, biolayer interferometry, ITC, isothermal titration calorimetry, mAb, monoclonal antibody, RBD, receptor-binding domain, RBM, receptor-binding motif, VOC, variant of concern, VOI, variant of interest, WT, wild-type

## Abstract

The seasonal nature of outbreaks of respiratory viral infections with increased transmission during low temperatures has been well established. Accordingly, temperature has been suggested to play a role on the viability and transmissibility of SARS-CoV-2, the virus responsible for the COVID-19 pandemic. The receptor-binding domain (RBD) of the Spike glycoprotein is known to bind to its host receptor angiotensin-converting enzyme 2 (ACE2) to initiate viral fusion. Using biochemical, biophysical, and functional assays to dissect the effect of temperature on the receptor–Spike interaction, we observed a significant and stepwise increase in RBD-ACE2 affinity at low temperatures, resulting in slower dissociation kinetics. This translated into enhanced interaction of the full Spike glycoprotein with the ACE2 receptor and higher viral attachment at low temperatures. Interestingly, the RBD N501Y mutation, present in emerging variants of concern (VOCs) that are fueling the pandemic worldwide (including the B.1.1.7 (α) lineage), bypassed this requirement. This data suggests that the acquisition of N501Y reflects an adaptation to warmer climates, a hypothesis that remains to be tested.

The etiological agent of the COVID-19 pandemic is the SARS-CoV-2 virus (1). While it will likely take years to understand the spread of SARS-CoV-2 infection in the human population, several factors could be modulating transmission dynamics and are currently being heavily scrutinized. As for other respiratory viruses, host immunity, population density, human behavioral factors, humidity, and temperature likely modulate its transmission (2, 3, 4, 5, 6, 7). Different steps in the replication cycle of coronaviruses could be affected by such factors, particularly viral entry. This process is mediated by the viral Spike (S) glycoprotein. The Spike glycoprotein uses its receptor-binding domain (RBD) to interact with its host receptor angiotensin-converting enzyme 2 (ACE2) (8, 9, 10). Cleavage by cell surface proteases or endosomal cathepsins (8, 11, 12) releases the fusion peptide and triggers irreversible conformational changes in the Spike glycoprotein enabling membrane fusion and viral entry (13, 14).

Airway transmission of SARS-CoV-2 is confronted to the temperature gradient that exists in human airways. In the nasal mucosa it is around 30 to 32 °C, it moves up to 32 °C in the upper trachea, and around 36 °C in the bronchi (15, 16). Emerging results strongly suggest that the Spike glycoprotein of SARS-CoV-2 evolved to replicate in the upper airways (17). This was linked to Spike stability, which was enhanced by the introduction of the D614G mutation (18, 19, 20) but also enhanced its use of cell surface and endosomal proteases (17, 21). Additionally, recent studies have noticed an increase replication of SARS-CoV-2 in primary human airway epithelial cells at 33 °C compared with 37 °C (22), while higher temperatures (39–40 °C) decreased overall viral replication (23). Since it was previously documented that temperature modulates the affinity of another viral envelope glycoprotein (HIV-1 Env) for its receptor (24); herein, we evaluate to what extent temperature affects the interaction of SARS-CoV-2 Spike with ACE2 and concomitantly, its effect on viral attachment.

## Results

### Conservation of ACE2-interacting residues among SARS-CoV-2 Spike isolates

**Figure 1:**
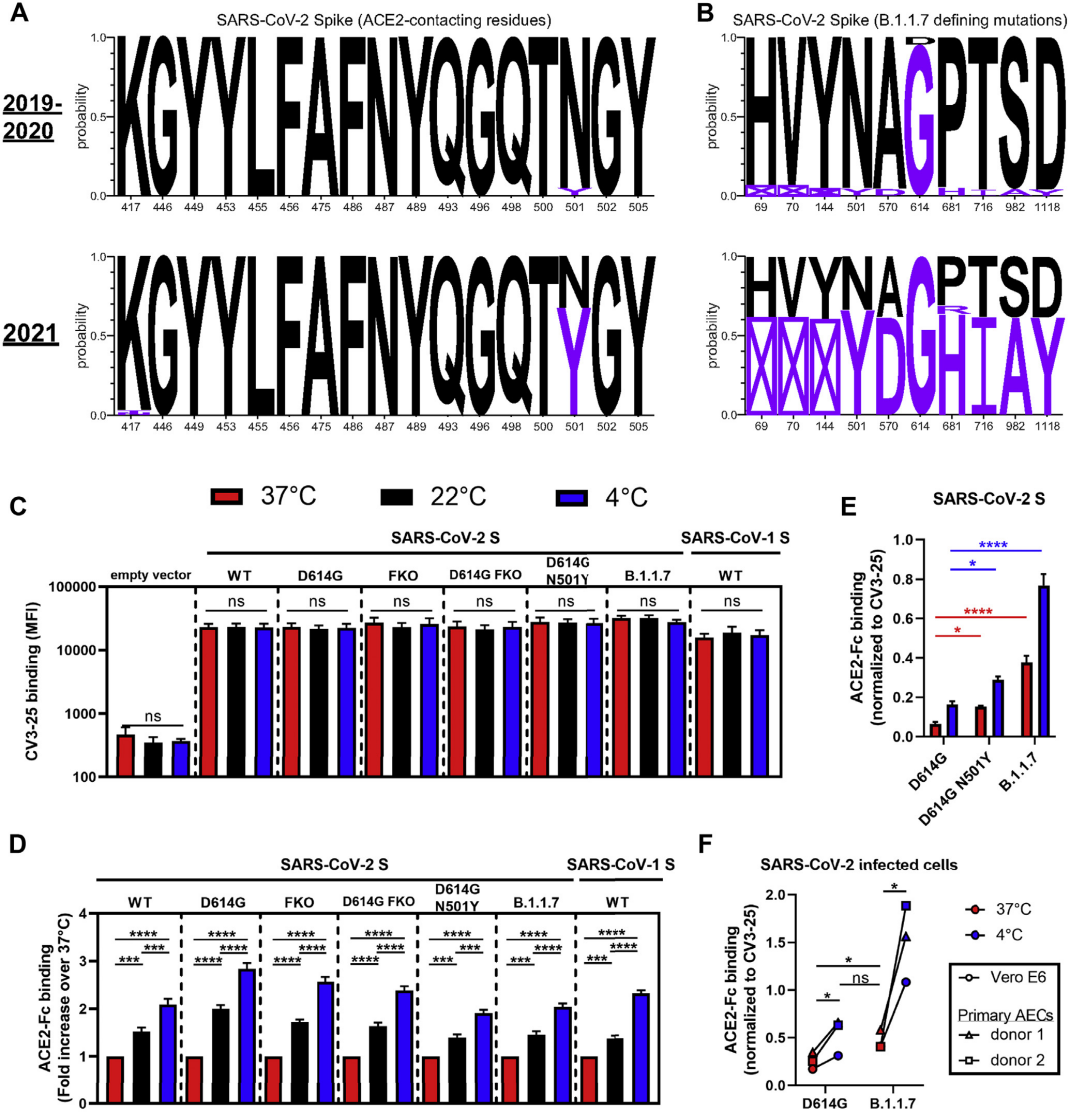
**Enhanced binding of ACE2 to SARS-CoV-2Spike at low temperatures.***A* and *B*, LOGO depictions of the frequency of SARS-CoV-2 Spike residues known to (*A*) contact with ACE2 or (*B*) corresponding to B.1.1.7 defining mutations. Worldwide sequences deposited in the GISAID database in 2019 to 2020 and in 2021 (up to June 18th, 2021) were aligned using the COVID CoV Genetics program. The height of the letter indicates its frequency over total sequences. Residues corresponding to the WIV04 reference sequence are shown in *black* and residues corresponding to VOCs are shown in *violet*. A *box with a cross inside* (☒) indicates the presence of a residue deletion. *C*–*E*, cell-surface staining of transfected 293T cells expressing SARS-CoV-2 Spike (WT, D614G, Furin KO, D614G Furin KO, D614G N501Y, or B.1.1.7 variant) or SARS-CoV-1 Spike (WT) using (*C*) CV3-25 mAb or (*D* and *E*) ACE2-Fc. *F*, cell-surface staining of Vero E6 or primary human AECs from two healthy donors infected with authentic SARS-CoV-2 viruses (D614G or B.1.1.7 variant) using ACE2-Fc. *C*–*F*, the graphs shown represent the binding of primary antibodies performed at (*C* and *D*) 37 °C, 22 °C, and 4 °C or (*E* and *F*) at 37 °C and 4 °C. ACE2-Fc binding was normalized to CV3-25 binding in each experiment at each temperature. The graphs shown represent the median fluorescence intensities (MFI). Error bars indicate means ± SEM. These results were obtained in at least three independent experiments. Statistical significance was tested using (*C*–*E*) one-way ANOVA with a Holm–Sidak posttest or (*F*) a paired *t* test (∗*p* < 0.05; ∗∗∗*p* < 0.001; ∗∗∗∗*p* < 0.0001; ns, nonsignificant).

We first evaluated the conservation of the ACE2-binding site on the SARS-CoV-2 Spike. Based on previously published structural data (25), ACE2 interacts with 17 key residues on the RBD primarily located on the receptor-binding motif (RBM). To determine the degree of conservation of these residues, we used the COVID-19 CoV Genetics program (26) and analyzed SARS-CoV-2 sequences deposited in 2019 to 2020 or in 2021 (up to June 18th 2021). Over the 2019 to 2020 period, no major sequence variations were observed except for the N501Y mutation (4.4%), which started to arise at the end of the year in at least three independent lineages of interest (B.1.1.7, B.1.351, P.1) (27, 28, 29). In 2021 sequences, most residues were still found to be >99% conserved except for variations found at residues 417 (K417N, 1.5%; K417T, 2.6%) and 501 (N501Y, 65.2%), with the latter becoming predominant among all the deposited sequences in 2021 (Fig. 1*A*). These mutations are found in emergent variants of concern (VOCs), including the B.1.1.7 (N501Y), B.1.351 (K417N/N501Y), and P.1 lineages (K417T/N501Y) (30). Among them, the B.1.1.7 lineage (also known as alpha variant), which was first identified in the United Kingdom, was shown to have increased infectivity and transmissibility (31, 32). This lineage spread rapidly and is the major circulating strain in early 2021 worldwide, replacing the D614G strain, which was predominant in 2019 to 2020 (Fig. 1*B*) (33). Sequence variations were also found in other RBM residues (Fig. S1), notably mutations L452R (8.8%), E484K (7.7%), T478K (5.9%), S477N (2.2%), and N439K (1.2%), which are also found in other various VOCs (34, 35, 36, 37, 38) and were shown to either increase infectivity or promote the evasion of antibody responses (30, 34, 38, 39, 40, 41, 42).

### Low temperatures increase SARS-CoV-2 Spike–ACE2 interaction

To measure the effect of temperature on the Spike–ACE2 interaction, we use a system where we express the full-length native Spike at the surface of cells and measure its interaction with the ACE2 receptor using a recombinant ACE2-Fc chimeric protein. This recombinant protein is composed of an ACE2 ectodomain linked to a human IgG1 Fc (43). 293T cells were transfected with a plasmid encoding the SARS-CoV-2 wild-type (WT) Spike (Wuhan-Hu-1 strain). Forty-eight hours posttransfection, cells were incubated at different temperatures (37 °C, 22 °C, and 4 °C) before measuring ACE2-Fc binding by flow cytometry. To ensure that any differential recognition was not linked to a temperature-dependent variation in Spike levels, we used the conformational-independent S2-targeting monoclonal antibody (mAb) CV3-25 as an experimental control (44, 45). As shown in Figure 1*C*, temperature did not alter CV3-25 recognition, indicating that temperature does not affect the overall amount of Spike at the surface of these cells. Therefore, the CV3-25 mAb was used to normalize Spike expression levels among the different mutants or variants (Fig. 1, *D*–*F*).

Interestingly, we observed a gradual increase in ACE2-Fc recognition concomitant with the temperature decrease (Fig. 1*D*), suggesting a temperature-dependent interaction between Spike and ACE2. Since temperature was suggested to also affect Spike stability (17, 46, 47), which in turn could explain its decreased receptor binding at 37 °C, we introduced the D614G change, known to increase trimer stability (17, 19) in combination or not with furin cleavage site mutations (FKO), known to prevent Spike proteolytic cleavage (48). The same stepwise increase in ACE2-Fc binding at lower temperatures was observed with all Spike constructs (Fig. 1*D*), indicating that low temperature can enhance ACE2-Fc binding independently of the strength of association between the S1 and S2 subunits. To extend these results to the Spike of emergent circulating strains, we evaluated ACE2-Fc binding to the Spike N501Y mutant and the Spike from the B.1.1.7 lineage. The N501Y mutation is located at the RBD-ACE2 interface and has been previously shown to strengthen the interaction with ACE2 by inserting an aromatic ring into a cavity at the binding interface (47, 48). Despite significantly higher binding of ACE2-Fc at 37 °C (Fig. 1*E*), a similar enhancement was observed with both the N501Y mutant and the B.1.1.7 variant at low temperatures (Fig. 1*D*). To evaluate whether this phenotype was conserved among other Betacoronaviruses, we also performed the same experiment using the closely related SARS-CoV-1 Spike and similar changes were observed (Fig. 1*D*).

To confirm our observations in a more physiological model, we infected a highly permissive cell line (Vero E6) and primary airway epithelial cells (AECs) from two different healthy donors using authentic SARS-CoV-2 virus isolated from patients infected with SARS-CoV-2 D614G or B.1.1.7 (Fig. 1*F*). Using flow cytometry, we discriminated the infected cells using an antinucleocapsid (N) mAb and measured the binding of ACE2-Fc at the cell surface (Fig. S2, *A* and *B*). We first noticed that the expression of trimeric Spike, as quantified by CV3-25 binding, is specific for the N+ population (Fig. S2, *A* and *B*). In agreement with results from transfected cells, the binding of ACE2 to cell surface Spike was higher at cold temperature (4 °C) compared with 37 °C for both D614G- and B.1.1.7-infected cells (Fig. 1*F* and Fig. S2*C*). Importantly, ACE2 bound to the B.1.1.7 Spike about two times more than to the D614G Spike at 37 °C (Fig. 1*F* and Fig. S2C). Similar level of binding could only be achieved for the D614G Spike by decreasing the temperature to 4 °C (Fig. 1*F*). Overall, low temperatures appear to promote Spike–ACE2 interaction independently of Spike trimer stability and emerging mutations, although the B.1.1.7 variant exhibited a pronounced improvement in binding at warmer temperatures.

### Low temperatures improve the viral attachment of SARS-CoV-2 virions

**Figure 2:**
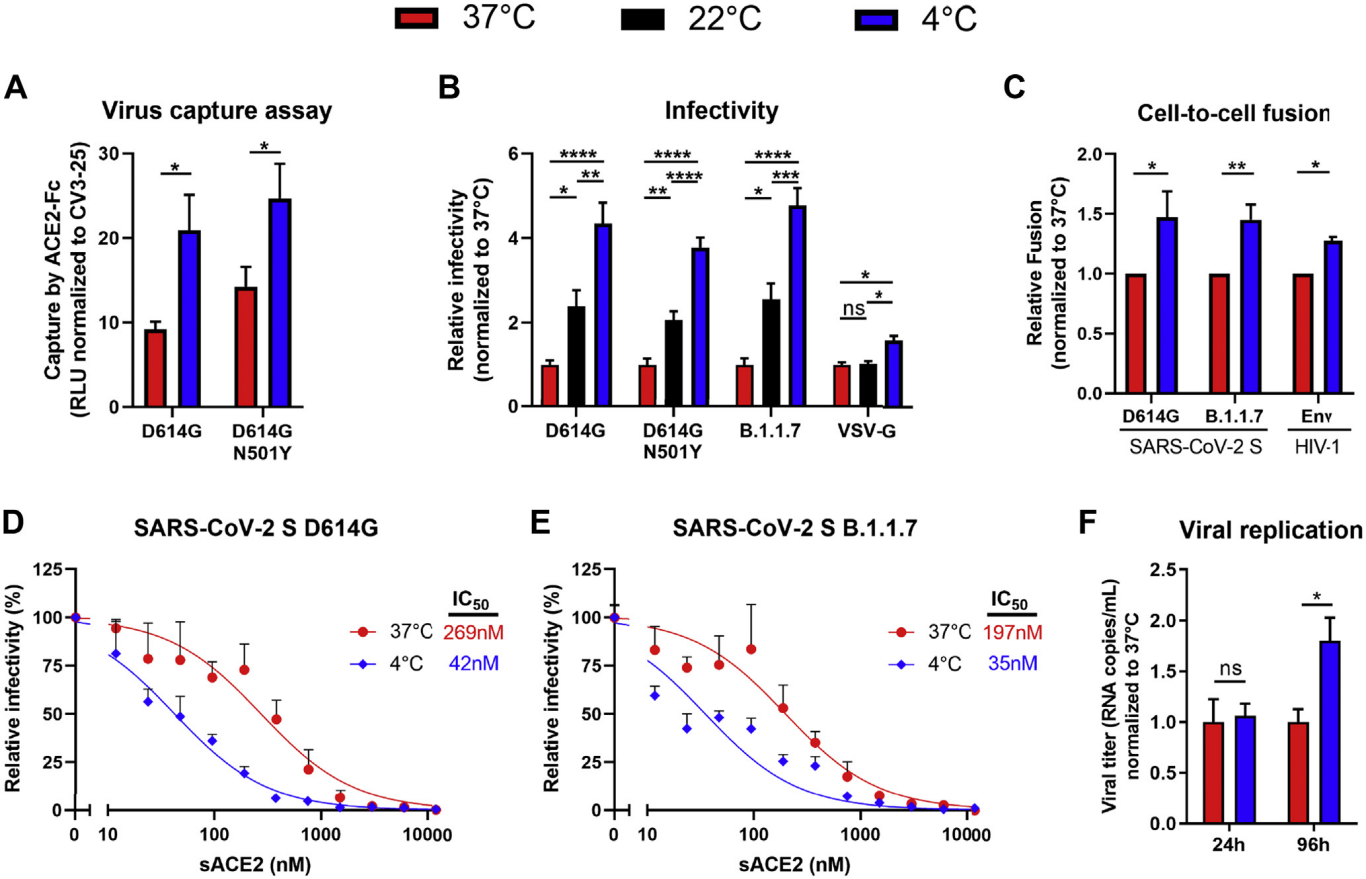
**SARS-CoV-2 viral attachment and infectivity is higher at low temperatures.***A*, pseudoviruses encoding the luciferase gene (Luc+) and bearing SARS-CoV-2 Spike (D614G or D614G N501Y) were tested for virus capture by ACE2-Fc at 37 °C or 4 °C. RLU obtained using ACE2-Fc was normalized to the signal obtained with the CV3-25 mAb. *B*, pseudoviruses Luc+ bearing SARS-CoV-2 Spike (D614G, D614G N501Y or B.1.1.7), or VSV-G as a control, were used to infect 293T-ACE2 cells. Virions were incubated at 37 °C, 22 °C, or 4 °C for 1 h prior infection of 293T-ACE2 cells for 48 h at 37 °C. *C*, cell-to-cell fusion was measured between 293T effector cells expressing HIV-1 Tat and SARS-CoV-2 Spike (D614G or B.1.1.7), or HIV-1 Env_JRFL_ as a control, which were incubated at 37 °C or 4 °C for 1 h prior coculture with TZM-bl-ACE2 target cells. *B* and *C*, RLUs were normalized to the signal obtained with cells preincubated at 37 °C. *D* and *E*, pseudoviruses Luc+ bearing SARS-CoV-2 Spike (WT, D614G or B.1.1.7) were used to infect 293T-ACE2 cells in presence of increasing concentrations of sACE2 at 37 °C for 1 h prior infection of 293T-ACE2 cells. Fitted curves and IC_50_ values were determined using a normalized nonlinear regression. *F*, authentic SARS-CoV-2 D614G virus was used to infect reconstituted human airway epithelia. Viral attachment was performed at 37 °C or 4 °C and cells were further cultured at 37 °C for 96 h. Viral titers (RNA copies/ml) were monitored at 24 h and 96 h postinfection using one-step qRT-PCR. Viral titer values were normalized to the signal obtained with virions adsorbed to the cells at 37 °C. Error bars indicate means ± SEM. These results were obtained in at least three independent experiments. Statistical significance was tested using (*A*, *C* and *F*) an unpaired *t* test or (*B*) one-way ANOVA with a Holm–Sidak post-test (∗*p* < 0.05; ∗∗*p* < 0.01; ∗∗∗*p* < 0.001; ∗∗∗∗*p* < 0.0001; ns, nonsignificant).

Next, we investigated the effect of enhanced ACE2 binding at low temperatures on SARS-CoV-2 Spike functional properties, including its ability to mediate viral attachment and fusion, and the subsequent consequences on early viral replication kinetics. To assess viral attachment, we adapted a previously described virus capture assay (49) where we generate lentiviral particles bearing SARS-CoV-2 Spike and look at their ability to interact with ACE2-Fc immobilized on ELISA plates. In agreement with a better affinity for ACE2 at lower temperatures, more SARS-CoV-2 D614G pseudoviral particles were captured at 4 °C compared with 37 °C (Fig. 2*A*). In line with these results, we also observed enhanced infectivity and cell-to-cell fusion mediated by SARS-CoV-2 Spike D614G at 4 °C compared with 37 °C, while a marginal increase was seen with an unrelated viral glycoprotein (VSV-G) (Fig. 2, *B* and *C*). In agreement with an enhanced affinity of HIV-1 Env for its receptor CD4 (24), HIV-1 Env-mediated fusion was also found to be temperature-dependent. Similarly, the capacity of soluble ACE2 (sACE2) to neutralize pseudovirions bearing SARS-CoV-2 Spike D614G was significantly improved when preincubating the virus with sACE2 at 4 °C when compared with 37 °C prior infection of 293T-ACE2 target cells (Fig. 2, *D* and *E*). Similar effects of temperature on Spike-mediated attachment and fusion and on sensitivity to sACE2 neutralization were observed when using the Spike N501Y mutant or B.1.1.7 variant (Fig. 2, *A*–*E*). To analyze the impact of temperature on viral replication in a more physiological model, we used authentic SARS-CoV-2 D614G viruses to infect reconstituted primary human airway epithelia (MucilAir). Infections were performed at 4 °C or 37 °C for 30 min, virus-containing medium was then discarded to remove any unbound virus before keeping the cells at 37 °C for 4 days. While no significant differences in viral titers were observed at 24 h postinfection, viral replication at 96 h postinfection was found to be significantly higher when the initial infection was performed at 4 °C *versus* 37 °C (Fig. 2*F*). Altogether, this suggests that lower temperatures improve the initial attachment of SARS-CoV-2, which in turn can alter the subsequent kinetics of viral replication.

### Low temperatures enhance the affinity of SARS-CoV-2 RBD for ACE2

**Figure 3:**
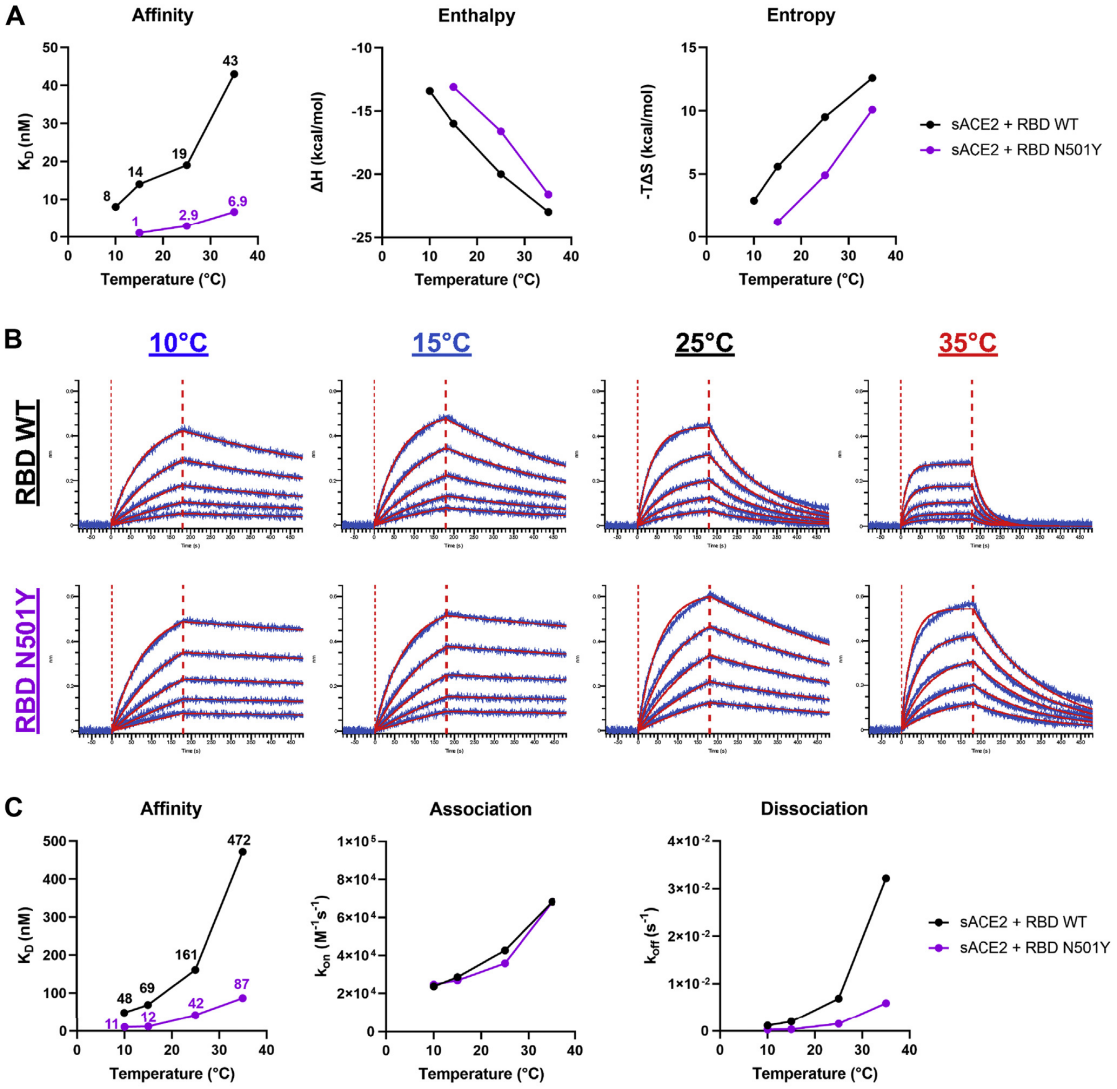
**Enhanced affinity of SARS-CoV-2 RBD for ACE2 at low temperatures.***A*, the thermodynamic parameters of sACE2 binding to SARS-CoV-2 RBD WT or N501Y measured by ITC at 10 °C, 15 °C, 25 °C, and 35 °C. The graphs shown represent the affinity (*K*_*D*_), enthalpy (*ΔH*), and entropy (*−TΔS*) values obtained at the different temperatures. All ITC titration curves and thermodynamics values are shown in Fig. S3. *B* and *C*, binding kinetics between SARS-CoV-2 RBD (WT or N501Y) and sACE2 assessed by BLI at different temperatures. *B*, biosensors loaded with RBD proteins were soaked in twofold dilution series of sACE2 (500 nM–31.25 nM) at different temperatures (10 °C, 15 °C, 25 °C, or 35 °C). Raw data are shown in *blue* and fitting model is shown in *red*. *C*, graphs represent the affinity constants (*K*_*D*_), on rates (*K*_*on*_) and off rates (*K*_*off*_) values obtained at different temperatures and calculated using a 1:1 binding model. All BLI data are summarized in Table S1.

We then evaluated whether the impact of temperature on ACE2 interaction could be recapitulated by the RBD alone. Isothermal titration calorimetry (ITC) was used to measure the binding of ACE2 to RBD at different temperatures ranging from 10 to 35 °C (Fig. S3*A*). The binding of ACE2 to RBD WT at 25 °C is characterized by a dissociation constant (*K*_*D*_) of 19 nM in a process that is associated with a favorable change in enthalpy of −20 kcal/mol, which is partially compensated by an unfavorable entropy contribution of 9.5 kcal/mol (Fig. 3*A* and Fig. S3*B*). The data obtained at different temperatures reveal a threefold increase in binding affinity from 43 nM at 35 °C to 14 nM at 15 °C. The observed effect of the temperature on *K*_*D*_ is expected based on the known temperature dependence of Gibbs energy of binding, *ΔG(T)*, which allows calculation of the expected *K*_*D*_ values at any temperature (Fig. S3*C*). Furthermore, as expected, the binding affinity of sACE2 to RBD N501Y was at least 6-fold higher than to RBD WT (Fig. 3*A*). The *K*_*D*_ value for the N501Y mutant is 2.9 nM at 25 °C and the respective values for the enthalpy and entropy contributions are −16.6 and 4.9 kcal/mol. The gain in affinity is the result of a loss in unfavorable entropy, which is larger than and overcompensates the loss in favorable enthalpy. Compared with ACE2 binding to RBD WT, the increase in binding affinity upon a drop in temperature is larger for the N501Y mutant with the *K*_*D*_ changing from 6.9 nM at 35 °C to 1 nM at 15 °C.

To better characterize how the temperature affects the binding kinetics between RBD and ACE2, we used biolayer interferometry (BLI) at the same temperatures as for ITC. RBD proteins were immobilized on biosensors and were soaked in increasing concentrations of sACE2, ranging from 31.25 to 500 nM (Fig. 3*B*). Again, affinity between RBD WT and sACE2 was found to be higher at lower temperatures (∼10-fold increase between 35 °C and 10 °C). Changes in affinity were explained by a major decrease in the off rate kinetics at low temperatures, despite a concomitant decrease in on-rate kinetics (Fig. 3*C* and Table S1). Compared with its WT counterpart, introduction of the N501Y mutation significantly decreased the off rate resulting in a 4.6-fold increase in *K*_*D*_ when performed at 25 °C. Remarkably, RBD WT reached a similar affinity for sACE2 at 10 °C than the one achieved by RBD N501Y at 25 °C (Fig. 3*B*). Altogether, this indicates that low temperatures or the N501Y mutation confers analogous affinity changes that are favorable for Spike RBD-ACE2 interaction.

### Low temperatures modulate SARS-CoV-2 Spike trimer opening

**Figure 4:**
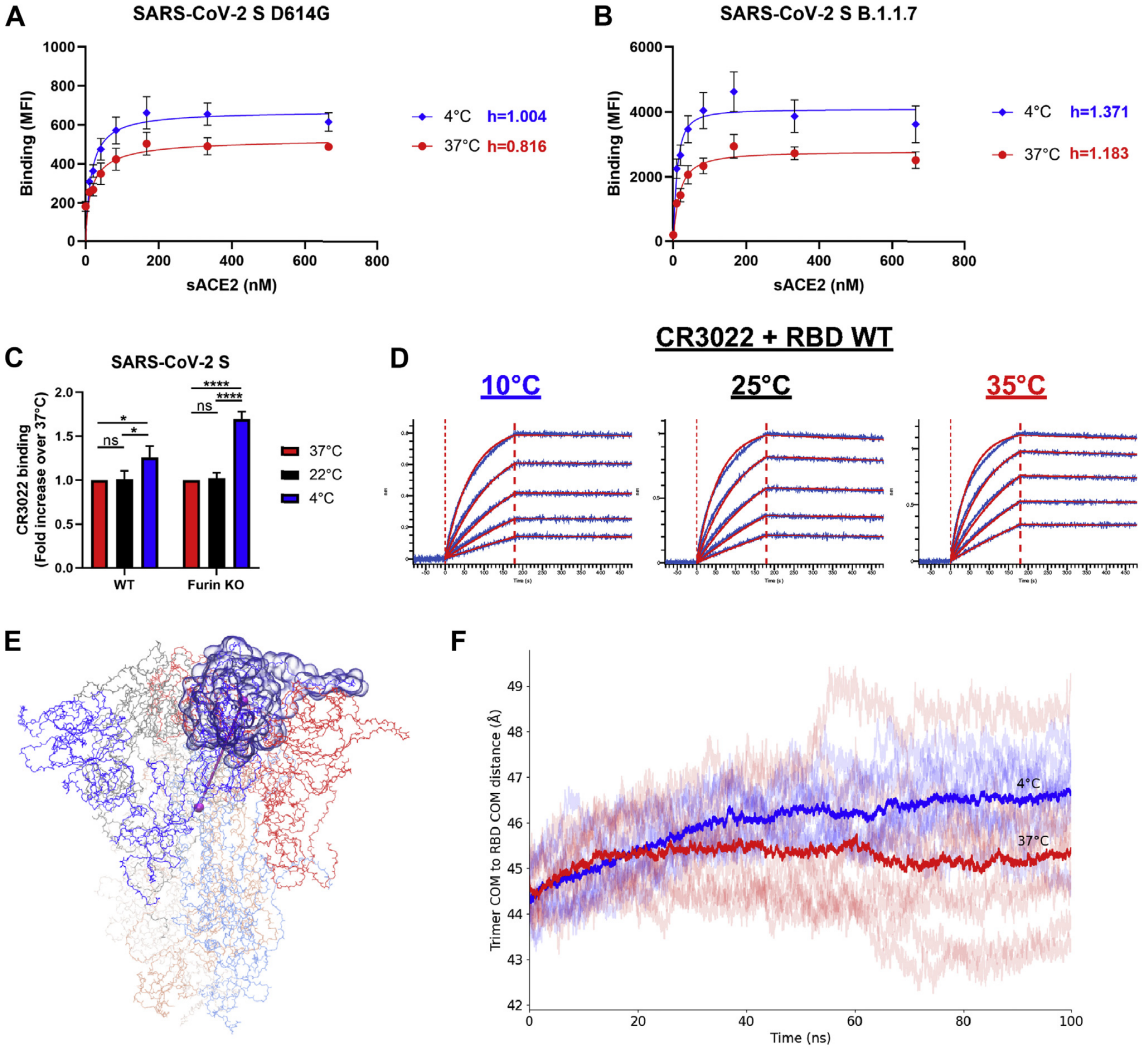
**SARS-CoV-2Spike trimer “opens” at low temperatures.***A* and *B*, binding of sACE2 to SARS-CoV-2 Spike (*A*) D614G or (*B*) B.1.1.7 expressed on 293T cells was measured at 37 °C or 4 °C by flow cytometry. Cells were preincubated with increasing amounts of sACE2 and its binding was detected using an anti-ACE2 staining. The Hill coefficients were determined using GraphPad software. *C*, cell-surface staining of transfected 293T cells expressing SARS-CoV-2 Spike (WT or Furin KO) using the CR3022 mAb when performed at 37 °C, 22 °C, or 4 °C. *A*–*C*, the graphs shown represent the median fluorescence intensities (MFI). Error bars indicate means ± SEM. These results were obtained in at least three independent experiments. Statistical significance was tested using (*C*) one-way ANOVA with a Holm–Sidak posttest (∗*p* < 0.05; ∗∗∗∗*p* < 0.0001; ns, nonsignificant). *D*, binding kinetics between RBD WT and CR3022 mAb assessed by BLI at 10 °C, 25 °C, or 35 °C. Biosensors loaded with RBD were soaked in twofold dilution series of CR3022 (100 nM–6.25 nM). Raw data are shown in *blue* and fitting model (1:1 binding model) is shown in *red*. All BLI data are summarized in Table S1. *E*, snapshot of SARS-CoV-2 Spike ectodomain (PDB 6VXX) (14) with one RBD indicated in transparent surface and one protomer’s RBD-to-trimer center-of-mass distance indicated with a cylinder. *F*, traces of the RBD-to-trimer distances from three replicas each of all-atom, fully glycosylated, and solvated MD simulations of the closed SARS-CoV-2 S trimer at 4 °C (*blues*) and 37 °C (*reds*) with dataset averages shown in heavy traces.

Since ACE2 interaction with Spike occurs when its RBD is in the “up” conformation (50, 51, 52), we sought to determine if temperature could also be modulating Spike trimer opening (*i.e.*, RBD accessibility). To do so, we evaluated the degree of cooperativity between sACE2 monomer binding within the Spike trimers by calculating the Hill coefficient (h), since ACE2 is thought to interact with Spike RBDs in a sequential manner (51). The h values are calculated from the steepness of dose–response curves generated upon incubation of Spike-expressing cells with increasing concentrations of sACE2 as previously described (43). We observed that the binding cooperativity of ACE2 to Spike D614G was slightly negative at 37 °C (h = 0.816), while being neutral at 4 °C (h = 1.004) (Fig. 4*A*). On the contrary, the binding cooperativity to Spike B.1.1.7 was already slightly positive at 37 °C (h = 1.183) and was further improved at 4 °C (h = 1.371), suggesting that B.1.1.7 mutations could facilitate a coordinated Spike opening in addition to its increased ACE2-RBD interaction, thus fueling the viral entry process (Fig. 4*B*). Spike conformational changes induced by temperature variation were also investigated by measuring the binding of the CR3022 mAb, which specifically recognizes the RBD “up” conformation (53, 54). Despite no clear change in binding affinity to RBD at low temperatures, CR3022 bound better to the membrane-bound trimeric Spike at 4 °C compared with 37 °C (Fig. 4, *C* and *D* and Table S1). Since CR3022 is known to disrupt prefusion Spike trimer (RBD) (53, 54), we also confirmed this phenotype using an uncleaved Spike version (Furin KO) (Fig. 4*C*). However, the increase in binding by CR3022 at 4 °C was minor compared with the one observed with ACE2-Fc and no change was seen at 22 °C, whereas ACE2-Fc binding was significantly higher (Figs. 1*D* and 4*C*). This confirms that low temperatures facilitate the exposure of the RBD in the “up” conformation, but it is unlikely sufficient on its own to recapitulate the temperature-dependent modulation of ACE2 interaction described in Figures 1 and 2.

To better understand how low temperature affects the conformational dynamics of Spike and the propensity of RBD to sample the “up” conformation, we performed all-atom molecular dynamics (MD) simulations to measure the distance between the center of mass of the trimer and the center of mass of each RBD subunit using the structure of a fully glycosylated closed SARS-CoV-2 Spike ectodomain trimer as a model (Fig. 4*E*) (14). Shown in Figure 4*F* is the RBD-to-trimer center distances of all S1 subunits in three replicas for each temperature (37 °C or 4 °C). At 4 °C, this distance is on average about 1.5 Å longer than at 37 °C, suggesting that lower temperatures favor conformations that are, on average, closer to RBD opening than do higher temperatures. This quaternary structural sensitivity to temperature is consistent with the observation that CR3022 is more reactive against full Spike trimers, but not RBD alone, at lower temperatures (Fig. 4, *C*–*F*).

## Discussion

In this study, we analyzed the role of temperature in modulating the affinity of SARS-CoV-2 Spike glycoprotein for its host receptor ACE2. We observed a significant enhancement in the affinity at low temperatures, which could be explained by favorable thermodynamics changes leading to a stabilization of the RBD-ACE2 interface and by the triggering of more “open” conformations of the Spike trimer. Consequently, SARS-CoV-2 entry events and early replication kinetics were found to be amplified by enhanced viral adsorption at cold temperatures. This could potentially lead to higher transmissibility and faster replication in upper airway tissues upon exposure to the virus at lower seasonal temperatures. Increasing evidence postulates for the establishment of a clear cycle of seasonal spread for SARS-CoV-2 infection once it reaches the endemic phase (55, 56), in a similar manner as other human coronaviruses and other respiratory viruses (57). Optimal air temperature for SARS-CoV-2 transmission has been suggested to range from 5 to 15 °C (7, 58). When entering the upper airways, such low temperature creates a gradient of temperature from the nasal cavity to the trachea, where it reaches around 33 °C (59, 60, 61). Combined with the fact that ACE2 is highly expressed in nasal epithelial cells (62), this makes the nasal cavity a remarkably favorable microenvironment for SARS-CoV-2 initial adsorption and early inoculum amplification (63), as previously observed (45, 64). While we did not explore this possibility, temperature could also be affecting viral replication kinetics postexposure and one could speculate that elevated body temperature resulting from SARS-CoV-2 infection (>38 °C) could participate in limiting virus replication *in vivo* by interfering with viral entry, as previously suggested (65).

In summary, our results suggest that the RBD from the original strain isolated in Wuhan requires lower temperature for optimal interaction with ACE2, whereas the N501Y mutation frees RBD from this requirement. A recent study compared a selection of SARS-CoV-2 Spike from emerging variants of concern (VOC) and variants of interest (VOI) for their sensitivity to cold temperature (66). The majority of them bound better to ACE2 at physiological temperature, notably for lineages harboring the N501Y mutation (B.1.1.7, B.1.351, and P.1) or the L452R mutation (B.1.617.2 and B.1.429). While all emerging variants bound better to ACE2 at low temperature, their sensitivity to cold temperature was less pronounced, especially for those harboring the N501Y mutation (66).

Whether this mechanism contributes to viral transmission and the apparent lack of seasonality for VOCs transmitted at warmer temperatures remains to be demonstrated. Our results indicate that the RBD-ACE2 affinity should be taken into consideration when evaluating the impact of the temperature on SARS-CoV-2 transmission.

## Experimental procedures

Experimental procedures are provided as supporting information.

## Data availability

All data are contained within the article.

## Supporting information

This article contains supporting information (8, 13, 14, 26, 43, 44, 45, 49, 67, 68, 69, 70, 71, 72, 73, 74, 75, 76, 77).

## Conflict of interest

The authors declare that they have no conflicts of interest with the contents of this article.

## Supporting information

Experimental procedures, Supplemental Figures S1–S3 and Table S1
