# Supplementary material for: Impact of temperature on the affinity of SARS-CoV-2 Spike glycoprotein for host ACE2
Source: J Biol Chem. 2021 Aug 31;297(4):101151. doi: 10.1016/j.jbc.2021.101151 (PMC8406544; doi:10.1016/j.jbc.2021.101151)
Supplement: Experimental procedures, Supplemental Figures S1–S3 and Table S1 [file mmc1.pdf]

Supporting information for

Impact of temperature on the affinity of SARS-CoV-2 Spike glycoprotein for host ACE2

Jérémie Prévost, Jonathan Richard, Romain Gasser, Shilei Ding, Clément Fage, Sai Priya Anand, Damien Adam, Natasha Gupta Vergara, Alexandra Tauzin, Mehdi Benlarbi, Shang Yu Gong, Guillaume Goyette, Anik Privé, Sandrine Moreira, Hugues Charest, Michel Roger, Walther Mothes, Marzena Pazgier, Emmanuelle Brochiero, Guy Boivin, Cameron F. Abrams, Arne Schön and Andrés Finzi

List of included material

Experimental Procedures

Figure S1. Sequence conservation of RBM residues among SARS-CoV-2 Spike isolates.

Figure S2. Flow cytometry analysis of SARS-CoV-2-infected cells staining.

Figure S3. Titration of sACE2 binding to SARS-CoV-2 RBD by isothermal titration calorimetry.

Table S1. Binding kinetics between SARS-CoV-2 RBD and its ligands quantified by biolayer interferometry.

EXPERIMENTAL PROCEDURES

Ethics statement

Primary airway epithelial cells (AECs) isolated from lung biopsies collected from healthy individuals were provided by the CRCHUM's Respiratory Cell and Tissue Biobank from the Respiratory Health Research Network of Québec with informed written consent prior to enrollment in accordance with Institutional Review Board approval (#CE08.063) and approval of the research study by the CRCHUM institutional review board (protocol #20.454). Research adhered to the standards indicated by the Declaration of Helsinki.

Sequence alignment

The Logo plots (1) were created using the WebLogo program (https://weblogo.berkeley.edu/logo.cgi) and the COVID-19 CoV Genetics interface (2) (https://covidcg.org/) using the GISAID database (https://www.gisaid.org/) to identify single-nucleotide polymorphism (SNP). The alignments correspond to the worldwide available sequences deposited between December 15th, 2019 and December 31st, 2020 (2019-2020 alignment) or January 1st, 2021 and June 18st, 2021 (2021 alignment), which includes 486,432 and 1,195,746 individual sequences of SARS-CoV-2 Spike RBD (residues 319-541), respectively. The relative height of each letter within individual stack represents the frequency of the indicated amino acid at that position. The numbering of all the Spike amino acid sequences is based on the prototypic WIV04 strain of SARS-CoV-2, where 1 is the initial methionine (3).

Cell lines, primary cells and viruses

293T human embryonic kidney cells (ATCC), Vero E6 african green monkey kidney cells (ATCC), Cf2Th (ATCC), 293T-ACE2 and TZM-bl-ACE2 cells were maintained at 37°C under 5% CO_2 in Dulbecco's Modified Eagle Medium (DMEM) (Wisent), supplemented with 5% fetal bovine serum (FBS) (VWR) and 100 U/mL penicillin/streptomycin (Wisent). 293T-ACE2 and TZM-bl-ACE2 cells stably expressing human ACE2 are derived from 293T cells and TZM-bl cells, respectively (4,5). TZM-bl cells are derived from HeLa cells and were engineered to contain the HIV-1 Tat-responsive firefly luciferase reporter gene. 293T-ACE2 and TZM-bl-ACE2 cells were then cultured in medium supplemented with 2 µg/mL of puromycin (Millipore Sigma). Cf2Th cells are SARS-CoV-2-resistant canine thymocytes and were used in the virus capture assay. Primary human airway epithelial cells (AECs) used in Figure 1F were isolated from bronchial biopsies collected from two healthy subjects (two males, mean age of 49 yrs). Briefly, bronchial tissues were rinsed and then incubated overnight at 4°C with MEM medium (Life Technologies) supplemented with 7.5% $NaHCO_3$ (Sigma-Aldrich), 2 mM L-glutamine, 10 mM HEPES (ThermoFisher Scientific), 0.05 mg/ml gentamycin, 50 U/ml penicillin-streptomycin, 0.25 µg/ml Fungizone (Life Technologies) and containing 0.1% protease (from Streptomyces griseus; Sigma-Aldrich) and 10 µg/ml DNAse (Deoxyribonuclease I from bovine pancreas; Sigma-Aldrich). The protease and DNAse activities were then neutralized with FBS (Life Technologies). AECs were gently scraped off the remaining tissue and red blood cells were removed by treatment with ACK lysis buffer (0.1 mM NH_4Cl, 10 µM $KHCO_3$, 10 nM EDTA). After counting, the freshly isolated cells were seeded into flasks coated with Purecol (Cedarlane) and collagen IV (Sigma-Aldrich), and grown in a mix (50:50) of PneumacultEx (STEMCELL Technologies) and CnT-17 (CellnTec Advanced Cell Systems) media for two days, and then in CnT-17 until the confluence is reached. AECs were then detached with a trypsin solution before

seeding into 6 wells plates coated with Purecol and collagen IV, cultured in CnT-17 until confluency (~5 days) ad then in BEGM (Lonza) for two days before experimentations. Authentic SARS-CoV-2 viruses used in Figure 1F were isolated, sequenced and amplified from clinical samples obtained from patients infected with SARS-CoV-2 D614G or B.1.1.7 by the Laboratoire de Santé Publique du Québec (LSPQ). SARS-CoV-2/Québec/CHUL/21697 is a clinical sample isolated in Quebec City, Canada and then amplified on Vero E6 cells. Virus was sequenced by MinION technology (Oxford Nanopore technologies, Oxford, UK). All work with infectious SARS-CoV-2 authentic virus was performed in a Biosafety Level 3 (BSL3) facilities at Université de Montréal and Université Laval using appropriate positive pressure air respirators and protective equipments.

Plasmids and site-directed mutagenesis

The plasmids expressing the wildtype SARS-CoV-2 and SARS-CoV-1 Spikes were previously reported (6,7). The plasmid encoding for SARS-CoV-2 S RBD (residues 319-541) fused with a hexahistidine tag was previously described (8). The mutations in full-length Spike (D614G, N501Y and/or R682S/R683S) or RBD (N501Y) expressors were introduced using the QuikChange II XL site-directed mutagenesis protocol (Agilent Technologies). The presence of the desired mutations was determined by automated DNA sequencing. The plasmid encoding the SARS-CoV-2 Spike from the B.1.1.7 lineage (Δ69-70, Δ144, N501Y, A570D, D614G, P681H, T716I, S982A and D1118H) was codon-optimized and synthesized by Genscript (9). The plasmid encoding for soluble human ACE2 (residues 1–615) fused with an 8xHisTag was reported elsewhere (10). The plasmid encoding for the ACE2-Fc chimeric protein, a protein composed of an ACE2 ectodomain (1–615) linked to an Fc segment of human IgG1 was

previously reported (11). The plasmids encoding for the vesicular stomatitis virus G (VSV-G) (pSVCMV-IN-VSV-G) and for the codon-optimized HIV-1 JRFL envelope glycoproteins were previously described (12,13).

Protein expression and purification

FreeStyle 293F cells (Thermo Fisher Scientific) were grown in FreeStyle 293F medium (Thermo Fisher Scientific) to a density of 1 x 10^6 cells/mL at 37°C with 8 % CO_2 with regular agitation (150 rpm). Cells were transfected with a plasmid coding for SARS-CoV-2 S RBD, sACE2 or ACE2-Fc using ExpiFectamine 293 transfection reagent, as directed by the manufacturer (Thermo Fisher Scientific). One week later, cells were pelleted and discarded. Supernatants were filtered using a 0.22 µm filter (Thermo Fisher Scientific). The recombinant RBD and sACE2 proteins were purified by nickel affinity columns, as directed by the manufacturer (Invitrogen). The ACE2-Fc chimeric protein was purified by protein A affinity columns, as directed by the manufacturer (Cytiva). The recombinant protein preparations were dialyzed against phosphate-buffered saline (PBS) and stored in aliquots at -80°C until further use. To assess purity, recombinant proteins were loaded on SDS-PAGE gels and stained with Coomassie Blue.

Flow cytometry analysis of cell-surface staining (transfected cells)

Using the standard calcium phosphate method, 10 µg of Spike expressor and 2 µg of a green fluorescent protein (GFP) expressor (pIRES2-eGFP; Clontech) was transfected into 2 × 10^6 293T cells. At 48h post transfection, 293T cells were stained with anti-Spike monoclonal antibodies CV3-25 (14) or CR3022 (15) (5 µg/mL) or using the ACE2-Fc chimeric protein (20 µg/mL) for 45 min at 37°C, 22°C or 4°C. Alternatively, to determine the Hill coefficients, cells were

preincubated with increasing concentrations of sACE2 (0 to 719 nM) at 37°C or 4°C. sACE2 binding was detected using a polyclonal goat anti-ACE2 (RND systems). Alexa Fluor-647-conjugated goat anti-human IgG (H+L) Abs (Invitrogen) and Alexa Fluor-647-conjugated donkey anti-goat IgG (H+L) Ab (Invitrogen) were used as secondary antibodies to stain cells for 30 min at room temperature. The percentage of transfected cells (GFP+ cells) was determined by gating the living cell population based on the basis of viability dye staining (Aqua Vivid, Invitrogen). Samples were acquired on a LSRII cytometer (BD Biosciences) and data analysis was performed using FlowJo v10.5.3 (Tree Star). Hill coefficient analyses were done using GraphPad Prism version 9.1.0 (GraphPad).

Flow cytometry analysis of cell-surface staining (infected cells)

SARS-CoV-2 authentic viruses (D614G or B.1.1.7 variant) were used to infect Vero E6 cells or primary AECs at a multiplicity of infection (MOI) of 0.0001 or 0.1, respectively. At 48h post-infection, cells were detached by PBS-EDTA and were stained with Abs for 30 min at 37°C or 4°C. Alexa Fluor-647-conjugated goat anti-human IgG (H+L) Ab (Invitrogen) was used as secondary antibody to stain cells for 30 min at room temperature. Cells were then fixed with PBS containing 4% paraformaldehyde for 48h at 4°C. Then the cells were stained intracellularly for SARS-CoV-2 nucleocapsid (N) antigen, using the Cytofix/Cytoperm fixation/permeabilization kit (BD Biosciences) and an anti-N mAb (clone mBG17; Kerafast) conjugated with the Alexa Fluor 488 dye according to the manufacturer's instructions (Invitrogen). The percentage of infected cells (N+ cells) was determined by gating the living cell population based on the basis of viability dye staining (Aqua Vivid, Invitrogen). Samples were acquired on a LSRII cytometer (BD Biosciences) and data analysis was performed using FlowJo v10.5.3 (Tree Star).

Virus capture assay

The SARS-CoV-2 virus capture assay was previously reported (16). Briefly, pseudoviral particles were produced by transfecting 2×10^6 293T cells with pNL4.3 R-E- Luc (3.5 μg), plasmids encoding for SARS-CoV-2 Spike (3.5 μg) proteins and VSV-G (1 μg) using the standard calcium phosphate method. Forty-eight hours later, virus-containing supernatants were collected, and cell debris were removed through centrifugation (1,500 rpm for 10 min). CV3-25 antibodies or ACE2-Fc proteins were immobilized on white MaxiSorp ELISA plates (Thermo Fisher Scientific) at a concentration of 5 μg/mL in 100 μL of PBS overnight at 4°C. Unbound proteins were removed by washing the plates twice with PBS. Plates were subsequently blocked with 3% bovine serum albumin (BSA) in PBS for 1 hour at room temperature, followed by 1 hour incubation at 37°C or 4°C. Meanwhile, virus-containing supernatants were pre-tempered at 37°C or 4°C for 1 hour. After washing plates twice with PBS, 200 μL of virus-containing supernatant were added to the wells. After 30 min of incubation at 37°C or 4°C, supernatants were discarded, and the wells were washed with PBS three times. Virus capture was visualized by adding 1×10^4 SARS-CoV-2-resistant Cf2Th cells per well in complete DMEM. Forty-eight hours post-infection, cells were lysed by the addition of 30 μL of passive lysis buffer (Promega) and one freeze-thaw cycle. An LB942 TriStar luminometer (Berthold Technologies) was used to measure the luciferase activity of each well after the addition of 100 μL of luciferin buffer (15 mM $MgSO_4$, 15 mM KH_2PO_4 [pH 7.8], 1 mM ATP, and 1 mM dithiothreitol) and 50 μL of 1 mM D-luciferin potassium salt (ThermoFisher Scientific).

Pseudovirus infectivity and neutralization assay

293T-ACE2 target cells were infected with single-round luciferase-expressing lentiviral particles (4). Briefly, 293T cells were transfected by the calcium phosphate method with the lentiviral vector pNL4.3 R-E- Luc (NIH AIDS Reagent Program) and a plasmid encoding for SARS-CoV-2 Spike or VSV-G at a ratio of 5:4. Two days post-transfection, cell supernatants were harvested and used fresh for infectivity measurements or stored at –80°C until use for virus neutralization measurements. 293T-ACE2 target cells were seeded at a density of 1×10^4 cells/well in 96-well luminometer-compatible tissue culture plates (Perkin Elmer) 24h before infection. To assess pseudovirus infectivity, freshly produced recombinant viruses were incubated for 1h at 37°C, 22°C or 4°C and were added to the target cells followed by incubation for 48h at 37°C. To measure virus neutralization by sACE2, recombinant viruses in a final volume of 100 µl were incubated with the increasing sACE2 concentrations (0 to 12,000 nM) for 1h at 37°C or 4°C and were then added to the target cells followed by incubation for 48h at 37°C; cells were lysed by the addition of 30 µl of passive lysis buffer (Promega) followed by one freeze-thaw cycle. An LB942 TriStar luminometer (Berthold Technologies) was used to measure the luciferase activity of each well after the addition of 100 µl of luciferin buffer (15 mM $MgSO_4$, 15 mM KH_2PO_4 [pH 7.8], 1 mM ATP, and 1 mM dithiothreitol) and 50 µl of 1 mM D-luciferin potassium salt (ThermoFisher Scientific). The neutralization half-maximal inhibitory dilution (IC_{50}) represents the sACE2 concentration inhibiting 50% of the infection of 293T-ACE2 cells by recombinant viruses bearing the indicated surface glycoproteins at different temperatures.

Cell-to-cell fusion assay

To assess the cell-to-cell fusion between Spike-expressing effector cells and ACE2-expressing target cells (5), 2×10^6 293T cells were co-transfected with plasmid expressing HIV-1 Tat (1 µg)

and a plasmid expressing SARS-CoV-2 Spike (4 µg) using the calcium phosphate method. Two days after transfection, Spike-expressing 293T (effector cells) were detached with PBS-EDTA 1 mM and incubated for 1 h at 37°C or 4°C. Subsequently, effector cells (1×10^4) were added to TZM-bl-ACE2 target cells that were seeded at a density of 1×10^4 cells/well in 96-well luminometer-compatible tissue culture plates 24 h before the assay. Cells were co-incubated for 6 h at 37°C and 5% CO_2, after which they were lysed by the addition of 40 µl of passive lysis buffer (Promega) and one freeze thaw cycle. An LB942 TriStar luminometer (Berthold Technologies) was used to measure the luciferase activity of each well after the addition of 100 µl of luciferin buffer (15 mM $MgSO_4$, 15 mM KH_2PO_4 [pH 7.8], 1 mM ATP, and 1 mM dithiothreitol) and 50 µL of 1 mM D-luciferin potassium salt (ThermoFisher Scientific).

Viral infection of reconstituted human airway epithelia (MucilAir)

For this experiment, an *ex vivo* system was used. This consists of an air liquid interface that mimicks the human upper airway epithelium. The primary nasal cells were isolated from a pool of 14 donors (MucilAir, Epithelix). The experiment was performed in quadruplicate and cells were cultured in medium provided by manufacturer. Before infection, MucilAir wells were washed with warm OptiMEM medium (ThermoFisher Scientific) and then were pre-incubated at 4°C or 37°C for 15 min. Apical poles were then infected directly with 200 µL of virus (SARS-CoV-2/Québec/CHUL/21697) at a multiplicity of infection (MOI) of 0.015 and then incubated at 4°C or 37°C during 30 min. After this adsorption phase, virus-containing medium was removed and all MucilAir wells were placed at 37°C. Samples were collected from apical washes (200 µL of OptiMEM) at different timepoints post-infection (24h and 96h) and 100 µL were used to extract RNA (MagNA Pure LC, Total nucleic acid isolation kit, Roche Applied Science). Viral

titers were determined by RT-qPCR one-step (QuantiTect Virus +ROX Vial Kit, Qiagen). To monitor the viability and health conditions of infected primary epithelial cells, transepithelial electrical resistance (TEER) was measured using a dedicated volt-ohm meter (Millicell® ERS-2, Millipore Sigma) and no significant difference was observed between cells infected at 4°C or 37°C.

Isothermal Titration Calorimetry (ITC)

Calorimetric titration experiments were performed using a MicroCal VP-ITC (Malvern Panalytical). The reagents were prepared in PBS pH 7.4, and then exhaustively dialyzed prior to the experiments. Any further dilutions of the reagents were made using the dialysate to avoid any unnecessary heats of dilution associated with the injections. All reagents were thoroughly degassed prior to the experiments. The enthalpy and affinity of binding at each temperature were determined from a complete titration of either RBD WT or the N501Y mutant with sACE2. The sACE2 solution at 11 - 13 µM was injected in 10 µL aliquots into the stirred calorimetric cell (v ~ 1.4 mL) containing RBD protein at ~ 1.5 µM. The titrations of RBD WT were performed at 10°C, 15°C, 25°C, and 35°C, while the titrations of the N501Y mutant were performed at 15°C, 25°C, and 35°C. The injection peaks were integrated, and the heat associated with binding was obtained after subtraction of the heats of dilution. The association constant, K_A (the dissociation constant, $K_D = 1/K_A$), the enthalpy change, ΔH, and the stoichiometry, N, were obtained by nonlinear regression of the data to a single-site binding model using Origin with a fitting function made in-house. Gibbs energy, ΔG, was calculated from the binding affinity using $\Delta G = -RT\ln K_A$, (R = 1.987 cal/(K × mol)) and T is the absolute temperature in kelvin). The entropy contribution to Gibbs energy, $-T\Delta S$, was calculated from the relation $\Delta G = \Delta H - T\Delta S$. The Gibbs

energy of binding, $\Delta G(T)$, as a known temperature dependence which allows calculation of the K_D values at any temperature according to:

$$\Delta G(T) = \Delta H_{ref} + \Delta C_p(T - T_{ref}) - T(\Delta S_{ref} + \Delta C_p \ln\left(\frac{T}{T_{ref}}\right)) \quad (1)$$

$$K_d = \frac{1}{e^{(\frac{-\Delta G}{RT})}} \quad (2)$$

where ΔH_{ref} and ΔS_{ref} are the respective enthalpy and entropy changes at the known reference temperature T_{ref}, T is the absolute temperature in kelvin, R the gas constant (1.987 cal/(K × mol)), and ΔC_p the change in heat capacity upon binding. The ΔC_p value for ACE2 binding is -380 cal/(K × mol) for RBD WT and -425 cal/(K × mol) for RBD N501Y, which is obtained from the temperature dependence of the enthalpy change (Fig 3A). Figure S2C shows a plot of the experimental dissociation constants at different temperatures together with the expected values calculated from ΔC_p and the enthalpy change at 25°C.

Biolayer interferometry (BLI)

Binding kinetics were performed on using an Octet RED96e system (ForteBio) at different temperatures (10°C, 15°C, 25°C, 35°C) with shaking at 1,000 RPM. Amine Reactive Second-Generation (AR2G) biosensors were hydrated in water, then activated for 300 s with a solution of 5 mM sulfo-NHS and 10 mM EDC (ForteBio) prior to amine coupling. Either SARS-CoV-2 RBD WT or the N501Y mutant were loaded into AR2G biosensor at 12.5 µg/mL at 25°C in 10 mM acetate solution pH 5 (Fortebio) for 600 s then quenched into 1 M ethanolamine solution pH 8.5 (Fortebio) for 300 s. Loaded biosensor were placed in 10X kinetics buffer (ForteBio) for 120 s for baseline equilibration. Association of sACE2 (in 10X kinetics buffer) to the different RBD proteins was carried out for 180 s at various concentrations in a two-fold dilution series from 500 nM to 31.25 nM prior to dissociation for 300 s. The data were baseline subtracted prior to fitting

performed using a 1:1 binding model and the ForteBio data analysis software. Calculation of on rates (k_{on}), off rates (k_{off}), and affinity constants (K_D) was computed using a global fit applied to all data. Alternatively, affinity of the CR3022 mAb at various concentrations in a two-fold dilution series from 100 nM to 6.25 nM for the immobilized SARS-CoV-2 RBD WT was assessed at different temperatures (10°C, 25°C, 35°C).

Molecular dynamics simulations

A fully glycosylated model of the closed SARS-CoV-2 S ectodomain trimer was built from the 6VXX PDB entry (17), a cryo-EM structure at a resolution of 2.80 Å of the Spike WT with all RBD in the down conformation. Six independent replicas with explicit waters were generated. Three were run for 100 ns at 37°C using NAMD 2.14 (18) (2 fs time step, Langevin thermostat with 5/ps frequency) and three were run for 100 ns at 4°C. The CHARMM36 force-field (19) and TIP3P water model were used. Configuration snapshots were saved every 2 ps. The instantaneous distance between the center of mass of the trimer and the center of mass of each receptor binding domain (residues 330 to 521 in each S1 subunit) was measured.

Statistical Analysis

Statistics were analyzed using GraphPad Prism version 9.1.0 (GraphPad, San Diego, CA, USA). Every data set was tested for statistical normality and this information was used to apply the appropriate (parametric or nonparametric) statistical test. P values <0.05 were considered significant; significance values are indicated as * P<0.05, ** P<0.01, *** P<0.001, **** P<0.0001.

REFERENCES

1. Crooks, G. E., Hon, G., Chandonia, J. M., and Brenner, S. E. (2004) WebLogo: a sequence logo generator. *Genome Res* **14**, 1188-1190
2. Chen, A. T., Altschuler, K., Zhan, S. H., Chan, Y. A., and Deverman, B. E. (2021) COVID-19 CG enables SARS-CoV-2 mutation and lineage tracking by locations and dates of interest. *Elife* **10**
3. Zhou, P., Yang, X. L., Wang, X. G., Hu, B., Zhang, L., Zhang, W., Si, H. R., Zhu, Y., Li, B., Huang, C. L., Chen, H. D., Chen, J., Luo, Y., Guo, H., Jiang, R. D., Liu, M. Q., Chen, Y., Shen, X. R., Wang, X., Zheng, X. S., Zhao, K., Chen, Q. J., Deng, F., Liu, L. L., Yan, B., Zhan, F. X., Wang, Y. Y., Xiao, G. F., and Shi, Z. L. (2020) A pneumonia outbreak associated with a new coronavirus of probable bat origin. *Nature* **579**, 270-273
4. Prevost, J., Gasser, R., Beaudoin-Bussieres, G., Richard, J., Duerr, R., Laumaea, A., Anand, S. P., Goyette, G., Benlarbi, M., Ding, S., Medjahed, H., Lewin, A., Perreault, J., Tremblay, T., Gendron-Lepage, G., Gauthier, N., Carrier, M., Marcoux, D., Piche, A., Lavoie, M., Benoit, A., Loungnarath, V., Brochu, G., Haddad, E., Stacey, H. D., Miller, M. S., Desforges, M., Talbot, P. J., Maule, G. T. G., Cote, M., Therrien, C., Serhir, B., Bazin, R., Roger, M., and Finzi, A. (2020) Cross-Sectional Evaluation of Humoral Responses against SARS-CoV-2 Spike. *Cell Rep Med* **1**, 100126
5. Ullah, I., Prevost, J., Ladinsky, M. S., Stone, H., Lu, M., Anand, S. P., Beaudoin-Bussieres, G., Symmes, K., Benlarbi, M., Ding, S., Gasser, R., Fink, C., Chen, Y., Tauzin, A., Goyette, G., Bourassa, C., Medjahed, H., Mack, M., Chung, K., Wilen, C. B., Dekaban, G. A., Dikeakos, J. D., Bruce, E. A., Kaufmann, D. E., Stamatatos, L., McGuire, A. T., Richard, J., Pazgier, M., Bjorkman, P. J., Mothes, W., Finzi, A., Kumar, P., and Uchil, P. D. (2021) Live imaging of SARS-CoV-2 infection in mice reveals that neutralizing antibodies require Fc function for optimal efficacy. *Immunity* **54**, 2143-2158 e2115
6. Hoffmann, M., Kleine-Weber, H., Schroeder, S., Kruger, N., Herrler, T., Erichsen, S., Schiergens, T. S., Herrler, G., Wu, N. H., Nitsche, A., Muller, M. A., Drosten, C., and Pohlmann, S. (2020) SARS-CoV-2 Cell Entry Depends on ACE2 and TMPRSS2 and Is Blocked by a Clinically Proven Protease Inhibitor. *Cell* **181**, 271-280 e278
7. Hoffmann, M., Muller, M. A., Drexler, J. F., Glende, J., Erdt, M., Gutzkow, T., Losemann, C., Binger, T., Deng, H., Schwegmann-Wessels, C., Esser, K. H., Drosten, C., and Herrler, G. (2013) Differential sensitivity of bat cells to infection by enveloped RNA viruses: coronaviruses, paramyxoviruses, filoviruses, and influenza viruses. *PLoS One* **8**, e72942
8. Beaudoin-Bussieres, G., Laumaea, A., Anand, S. P., Prevost, J., Gasser, R., Goyette, G., Medjahed, H., Perreault, J., Tremblay, T., Lewin, A., Gokool, L., Morrisseau, C., Begin, P., Tremblay, C., Martel-Laferriere, V., Kaufmann, D. E., Richard, J., Bazin, R., and Finzi, A. (2020) Decline of Humoral Responses against SARS-CoV-2 Spike in Convalescent Individuals. *mBio* **11**
9. Tauzin, A., Nayrac, M., Benlarbi, M., Gong, S.Y., Gasser, R., Beaudoin-Bussières, G., Brassard, N., Laumaea, A., Vézina, D., Prévost, J., Anand, S.P., Bourassa, C., Gendron-Lepage, G., Medjahed, H., Goyette, G., Niessl, J., Tastet, O., Gokool, L., Morrisseau, C., Arlotto, P., Stamatatos, L., McGuire, A.T., Larochelle, C., Uchil, P., Lu, M., Mothes, W., De Serres, G., Moreira, S., Roger, M., Richard, J., Martel-Laferrière, V., Duerr, R.,

Tremblay, C., Kaufmann, D.E., Finzi, A. . (2021) A single BNT162b2 mRNA dose elicits antibodies with Fc-mediated effector functions and boost pre-existing humoral and T cell responses. *Cell Host Microbe* **29**, 1137-1150 e1136

10. Wrapp, D., Wang, N., Corbett, K. S., Goldsmith, J. A., Hsieh, C. L., Abiona, O., Graham, B. S., and McLellan, J. S. (2020) Cryo-EM structure of the 2019-nCoV spike in the prefusion conformation. *Science* **367**, 1260-1263
11. Anand, S. P., Chen, Y., Prevost, J., Gasser, R., Beaudoin-Bussieres, G., Abrams, C. F., Pazgier, M., and Finzi, A. (2020) Interaction of Human ACE2 to Membrane-Bound SARS-CoV-1 and SARS-CoV-2 S Glycoproteins. *Viruses* **12**
12. Lodge, R., Lalonde, J. P., Lemay, G., and Cohen, E. A. (1997) The membrane-proximal intracytoplasmic tyrosine residue of HIV-1 envelope glycoprotein is critical for basolateral targeting of viral budding in MDCK cells. *EMBO J* **16**, 695-705
13. Mao, Y., Wang, L., Gu, C., Herschhorn, A., Xiang, S. H., Haim, H., Yang, X., and Sodroski, J. (2012) Subunit organization of the membrane-bound HIV-1 envelope glycoprotein trimer. *Nat Struct Mol Biol*
14. Jennewein, M. F., MacCamy, A. J., Akins, N. R., Feng, J., Homad, L. J., Hurlburt, N. K., Seydoux, E., Wan, Y. H., Stuart, A. B., Edara, V. V., Floyd, K., Vanderheiden, A., Mascola, J. R., Doria-Rose, N., Wang, L., Yang, E. S., Chu, H. Y., Torres, J. L., Ozorowski, G., Ward, A. B., Whaley, R. E., Cohen, K. W., Pancera, M., McElrath, M. J., Englund, J. A., Finzi, A., Suthar, M. S., McGuire, A. T., and Stamatatos, L. (2021) Isolation and characterization of cross-neutralizing coronavirus antibodies from COVID-19+ subjects. *Cell Rep* **36**, 109353
15. ter Meulen, J., van den Brink, E. N., Poon, L. L., Marissen, W. E., Leung, C. S., Cox, F., Cheung, C. Y., Bakker, A. Q., Bogaards, J. A., van Deventer, E., Preiser, W., Doerr, H. W., Chow, V. T., de Kruif, J., Peiris, J. S., and Goudsmit, J. (2006) Human monoclonal antibody combination against SARS coronavirus: synergy and coverage of escape mutants. *PLoS Med* **3**, e237
16. Ding, S., Laumaea, A., Benlarbi, M., Beaudoin-Bussieres, G., Gasser, R., Medjahed, H., Pancera, M., Stamatatos, L., McGuire, A. T., Bazin, R., and Finzi, A. (2020) Antibody Binding to SARS-CoV-2 S Glycoprotein Correlates with but Does Not Predict Neutralization. *Viruses* **12**
17. Walls, A. C., Park, Y. J., Tortorici, M. A., Wall, A., McGuire, A. T., and Veesler, D. (2020) Structure, Function, and Antigenicity of the SARS-CoV-2 Spike Glycoprotein. *Cell* **181**, 281-292 e286
18. Phillips, J. C., Braun, R., Wang, W., Gumbart, J., Tajkhorshid, E., Villa, E., Chipot, C., Skeel, R. D., Kale, L., and Schulten, K. (2005) Scalable molecular dynamics with NAMD. *J Comput Chem* **26**, 1781-1802
19. MacKerell, A. D., Bashford, D., Bellott, M., Dunbrack, R. L., Evanseck, J. D., Field, M. J., Fischer, S., Gao, J., Guo, H., Ha, S., Joseph-McCarthy, D., Kuchnir, L., Kuczera, K., Lau, F. T., Mattos, C., Michnick, S., Ngo, T., Nguyen, D. T., Prodhom, B., Reiher, W. E., Roux, B., Schlenkrich, M., Smith, J. C., Stote, R., Straub, J., Watanabe, M., Wiorkiewicz-Kuczera, J., Yin, D., and Karplus, M. (1998) All-atom empirical potential for molecular modeling and dynamics studies of proteins. *J Phys Chem B* **102**, 3586-3616

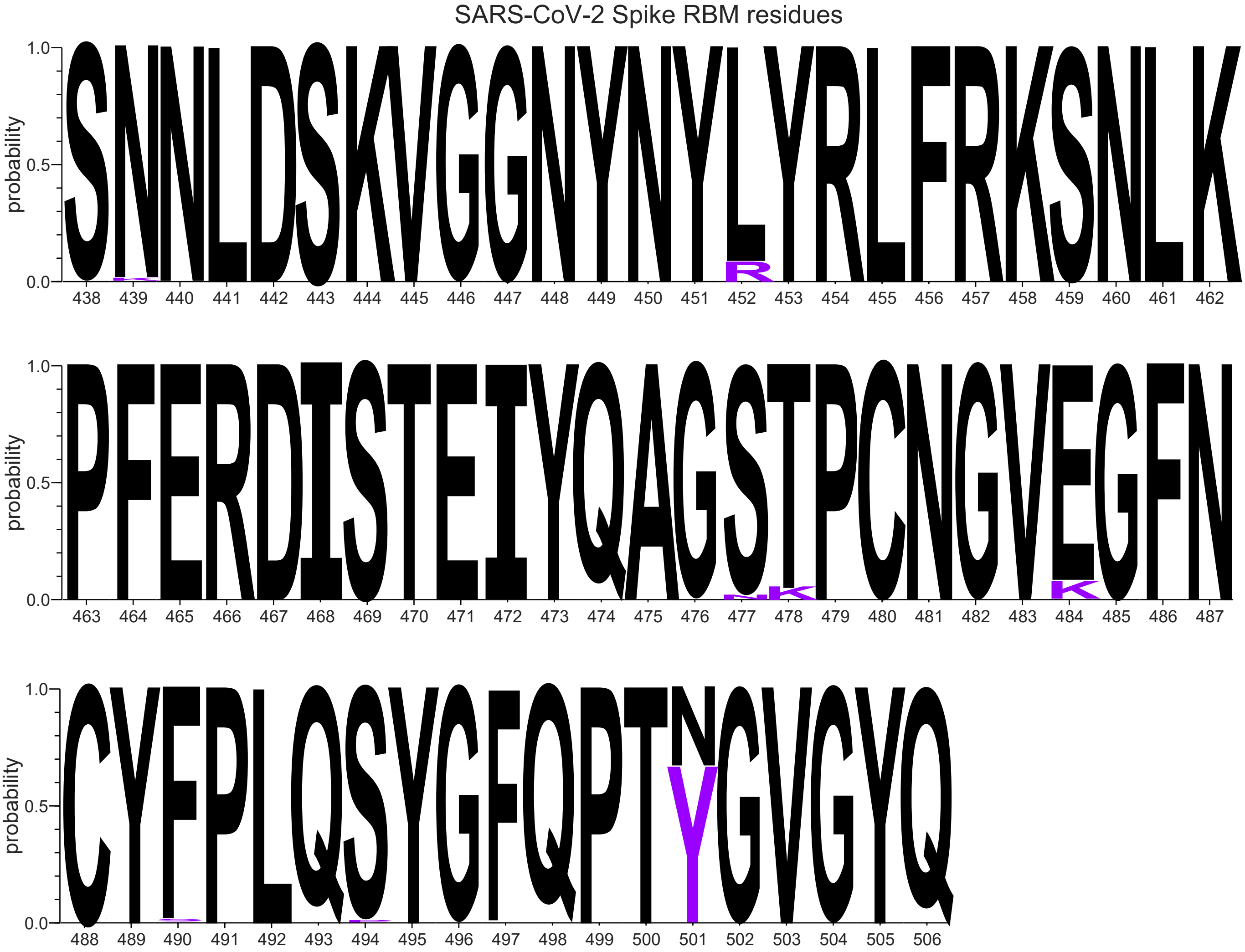

Figure S1. Sequence conservation of RBM residues among SARS-CoV-2 Spike isolates. Logo depictions of the frequency of selected SARS-CoV-2 Spike residues from the receptor binding motif (RBM). Worldwide sequences deposited in the GISAID database in 2021 (January 1st, 2021 to June 18th, 2021) were aligned using the COVID CoV Genetics program, which includes 1,195,746 individual sequences. Residue numbering is based on the SARS-CoV-2 WIV04 reference strain. The height of the letter indicates its frequency of total deposited sequences. Residues corresponding to the WIV04 reference sequence are shown in black and residues corresponding to emerging VOCs are shown in violet.

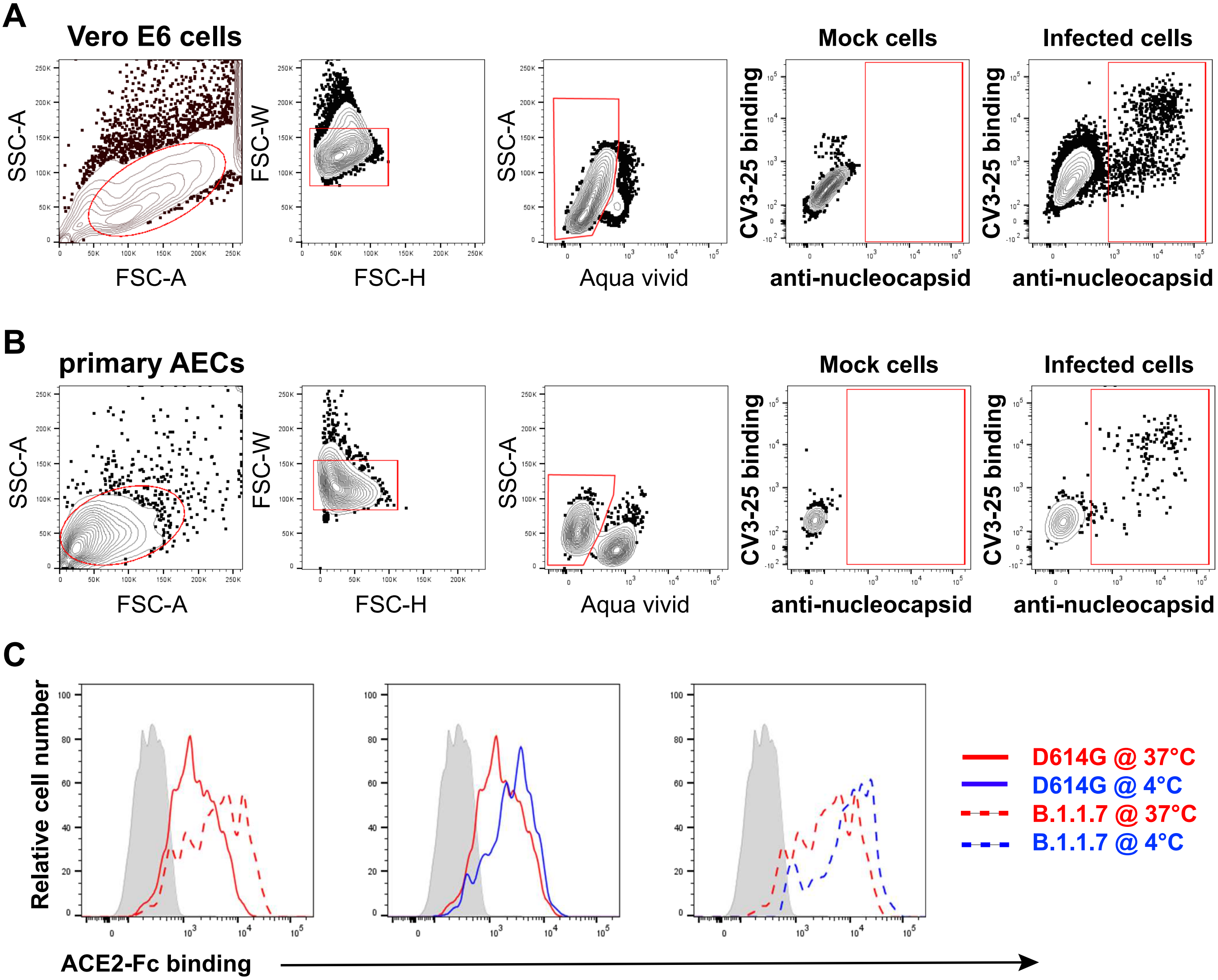

Figure S2. Flow cytometry analysis of SARS-CoV-2-infected cells staining. Representative flow cytometry gates of (**A**) Vero E6 or (**B**) primary human AECs infected with an authentic SARS-CoV-2 virus for the cell-surface staining with CV3-25 and the intracellular staining against the nucleocapsid viral antigen (N). The histograms shown in (**C**) represent the binding of ACE2-Fc on the N+ population from D614G- or B.1.1.7-infected Vero E6 cells (or mock-infected cells in gray shaded curves) performed at 37°C or 4°C.

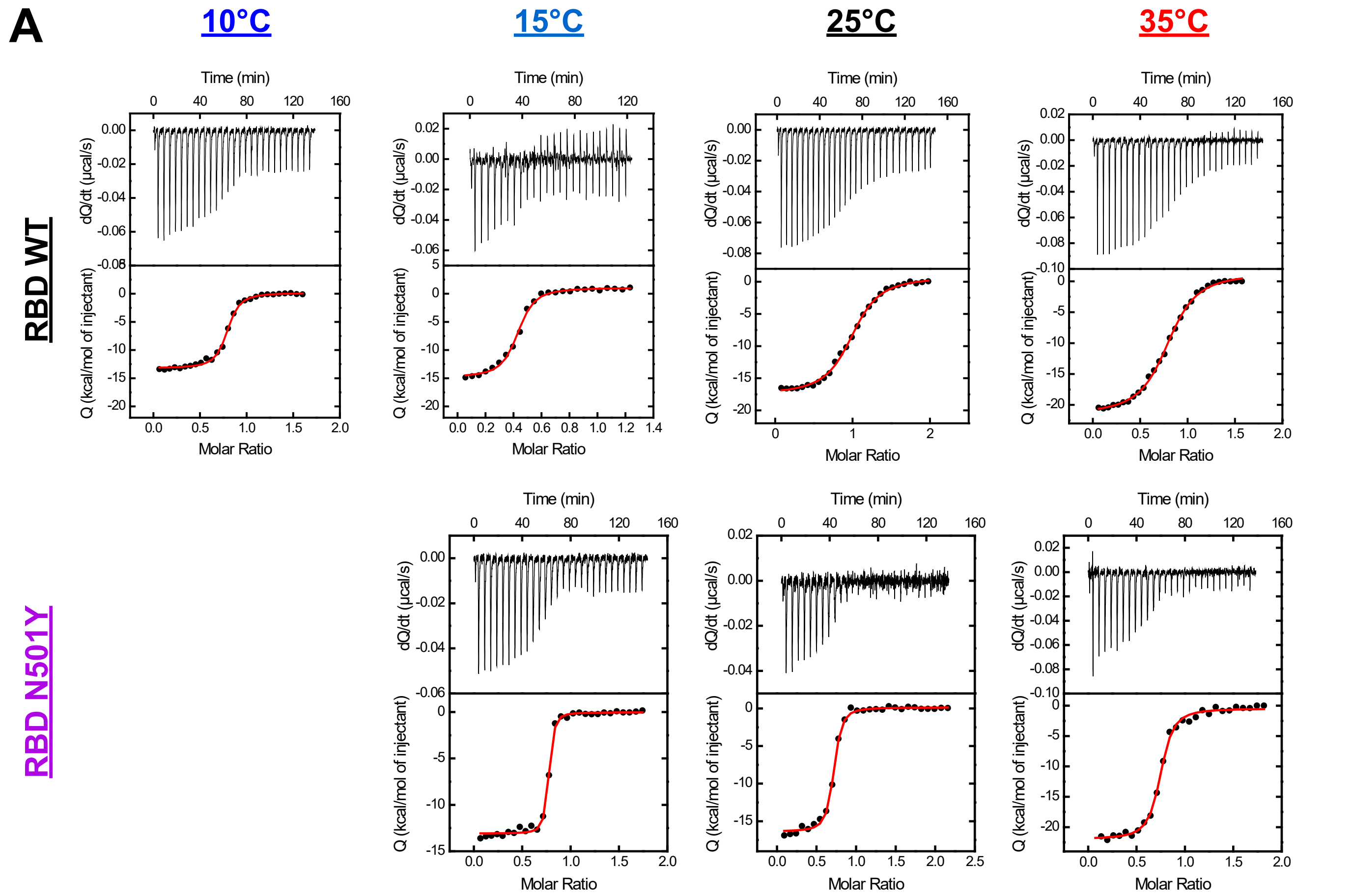

B

Ligands	Temperature	K_D (nM)	ΔG (kcal/mol)	ΔH (kcal/mol)	-TΔS (kcal/mol)
sACE2 + RBD WT	10°C	8	-10.5	-13.4	+2.9
	15°C	14	-10.4	-16.0	+5.6
	25°C	19	-10.5	-20.0	+9.5
	35°C	43	-10.4	-23.0	+12.6
sACE2 + RBD N501Y	15°C	1	-11.9	-13.1	+1.2
	25°C	2.9	-11.7	-16.6	+4.9
	35°C	6.7	-11.5	-21.6	+10.1

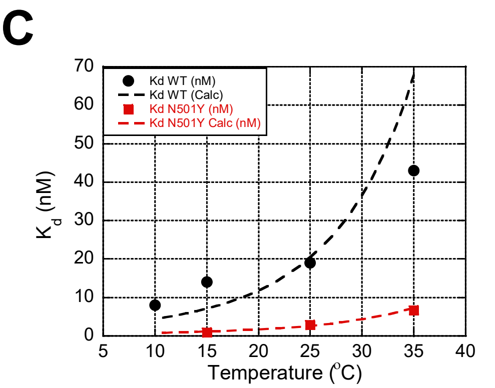

Figure S3. Titration of sACE2 binding to SARS-CoV-2 RBD by isothermal titration calorimetry. (**A**) ITC data from calorimetric titrations of SARS-CoV-2 RBD (WT and N501Y) with sACE2 at different temperatures. The top panel of each graph shows the heat flow, dQ/dt, as a function of time and the bottom panel shows the integrated heat associated with each injection (Q) as a function of the molar ratio between the concentrations of sACE2 and RBD in the cell. The solid line represents the result from best nonlinear least squares fit of the data to a single-site binding model. (**B**) Table summarizing the thermodynamic parameters for the binding of sACE2 to SARS-CoV-2 RBD (WT and N501Y). The values for the dissociation constant, K_D, and enthalpy, *ΔH*, were obtained directly from nonlinear regression of the ITC data (**A**), while Gibbs energy, *ΔG*, and the entropy contribution, *-TΔS*, were calculated as described in the method section. (**C**) The K_D values for the binding of sACE2 to SARS-CoV-2 RBD WT (filled circles) and N501Y (filled squares) as a function of temperature. The dashed lines correspond to the expected values calculated from ΔC_p and the enthalpy change at 25°C.

Table S1. Affinity between SARS-CoV-2 RBD and its ligands quantified by biolayer interferometry.

Ligands	Temperature	K_D (nM)	K_{on} ($M^{-1}s^{-1}$)	K_{off} (s^{-1})
sACE2 + RBD WT	10°C	47.6	2.37×10^4	1.13×10^{-3}
	15°C	68.5	2.86×10^4	1.96×10^{-3}
	25°C	161	4.27×10^4	6.88×10^{-3}
	35°C	472	6.83×10^4	3.22×10^{-2}
sACE2 + RBD N501Y	10°C	10.7	2.47×10^4	2.65×10^{-4}
	15°C	11.9	2.69×10^4	3.19×10^{-4}
	25°C	41.5	3.59×10^4	1.49×10^{-3}
	35°C	86.5	6.83×10^4	5.91×10^{-3}
CR3022 + RBD WT	10°C	0.124	1.51×10^5	1.87×10^{-5}
	25°C	0.649	1.77×10^5	1.15×10^{-4}
	35°C	0.457	2.03×10^5	9.26×10^{-5}
